# The Visualization of Nocturnal Scratching Behavior in Pediatric Atopic Dermatitis: Facilitating Shared Decision-Making and Assessing the Efficacy of Treatment

**DOI:** 10.7759/cureus.88988

**Published:** 2025-07-29

**Authors:** Akiko Sugiyama, Hiroshi Matsuzaki, Chikako Motomura, Tetsuya Hiramoto, Takeshi Nakahara

**Affiliations:** 1 Department of Allergology, National Hospital Organization Fukuoka National Hospital, Fukuoka, JPN; 2 Department of Pediatrics, National Hospital Organization Fukuoka National Hospital, Fukuoka, JPN; 3 Department of Clinical Research, National Hospital Organization Fukuoka National Hospital, Fukuoka, JPN; 4 Dermatology, Kyushu University, Fukuoka, JPN

**Keywords:** atopic dermatitis, itch tracker, nemolizumab, scratching, shared decision-making

## Abstract

Itching is a subjective symptom that is especially difficult to assess in pediatric atopic dermatitis (AD) patients with chronic pruritus since early infancy. This study aimed to assess the utility of the Itch Tracker (Maruho Co. Ltd., Osaka, Japan), a smartwatch application, in visualizing nocturnal scratching and facilitating shared decision-making (SDM) in two pediatric AD cases treated with nemolizumab. Objective monitoring revealed discrepancies between self-reported itch and actual scratching behavior, leading to appropriate treatment initiation and significant improvements in scratching behavior, Eczema Area and Severity Index (EASI), and numerical rating scale (NRS) scores. These results highlight the clinical value of behavioral visualization in enhancing SDM and optimizing management in pediatric AD.

## Introduction

Itching, defined as an “unpleasant sensation that makes one want to scratch,” is inherently subjective and difficult to quantify [1-3]. In pediatric atopic dermatitis (AD), where patients often have a lifelong history of pruritus, many have never experienced an itch-free state [3]. This makes self-assessment tools like the numerical rating scale (NRS) or visual analog scale (VAS) less reliable [4,5]. Some patients may underestimate their pruritus due to habituation, whereas others may exaggerate it as a result of increased sensitivity or psychological stress [6,7]. Since scratching can worsen inflammation, evaluating the itch is crucial. This study presents two pediatric cases where nocturnal scratching behavior was visualized using Itch Tracker (Maruho Co. Ltd., Osaka, Japan), a smartwatch-based application designed to analyze acceleration data from the Apple Watch (Apple Inc., Cupertino, CA) to objectively measure nocturnal scratching. Previous studies have reported that the application is a useful tool for observing nocturnal scratching behavior in children with AD [1], facilitating shared decision-making (SDM). The two cases presented in this study involved observation of scratching behavior before and after the administration of nemolizumab. Visualization of scratching behavior enabled SDM with the patients regarding the treatment strategy.

## Case presentation

Case 1

**Figure 1:**
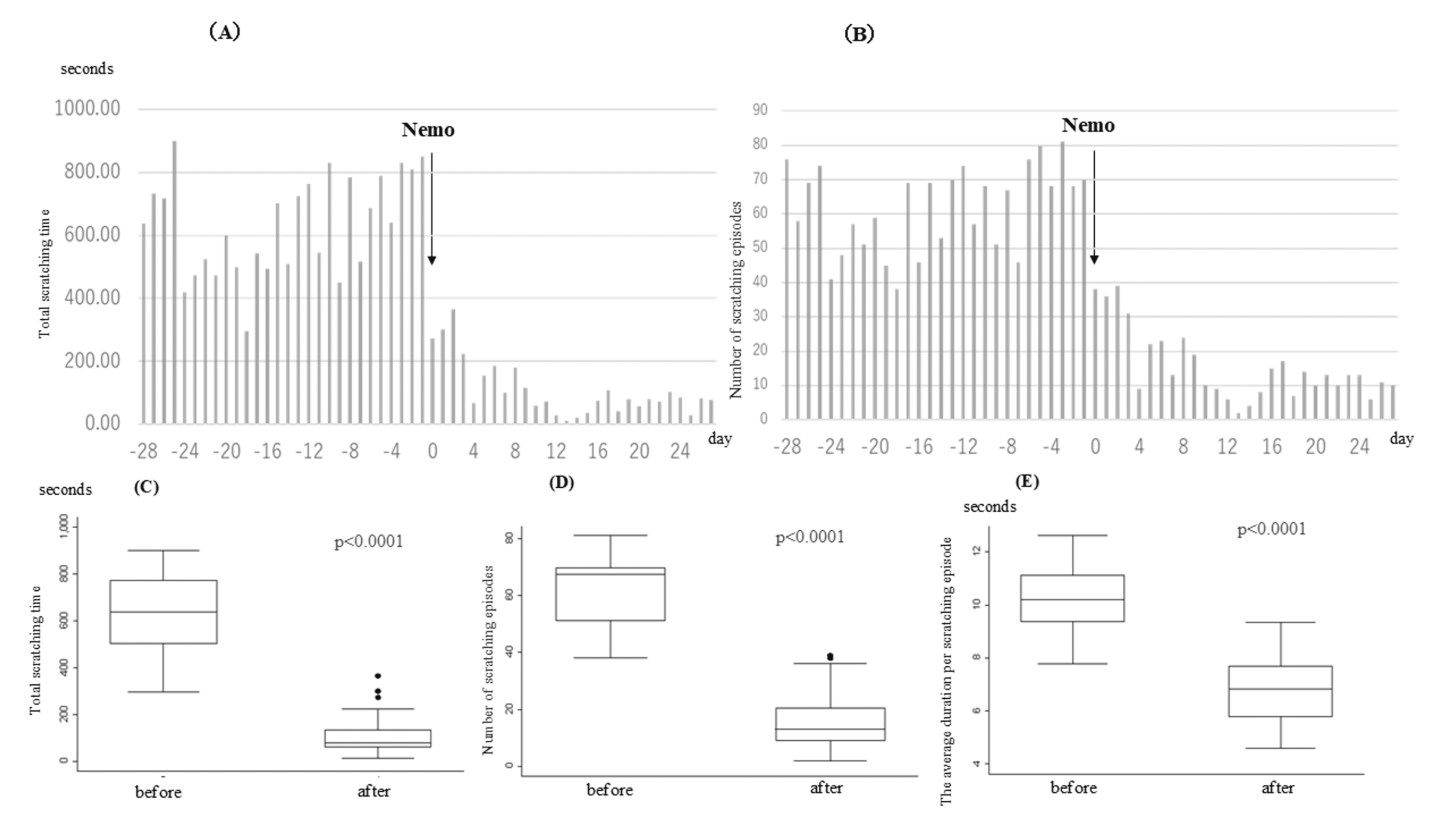
Changes in nocturnal scratching behavior before and after nemolizumab administration (case 1) (A) Daily total scratching time (in seconds) and (B) the number of scratching episodes observed over 28 days before and after the administration of Nemo (nemolizumab). The day of Nemo initiation is designated as Day 0. Scratching behavior decreased after treatment. (C) Box plots showing the total scratching time, (D) the number of scratching episodes, and (E) the average duration per scratching episode before and after Nemo administration. All metrics significantly improved (p<0.0001)

A 15-year-old male with a history of AD since infancy presented with persistent nocturnal scratching according to the family despite moderate skin inflammation. He had been treated with topical therapies since early childhood, and during the past year, his treatment mainly consisted of strong or very strong-class topical corticosteroids. The patient denied severe itching, but his family reported frequent nighttime scratching. Scratching behavior was recorded for one month, confirming frequent episodes. The dosing of nemolizumab 60 mg was initiated, with an Eczema Area and Severity Index (EASI) score of 16.6 and NRS of 6. At the time of the second dose (day 28), EASI had decreased to 3.6 and NRS to 3. Scratching frequency, total duration, and mean episode length all significantly decreased post-treatment (Figure 1).

Case 2

**Figure 2:**
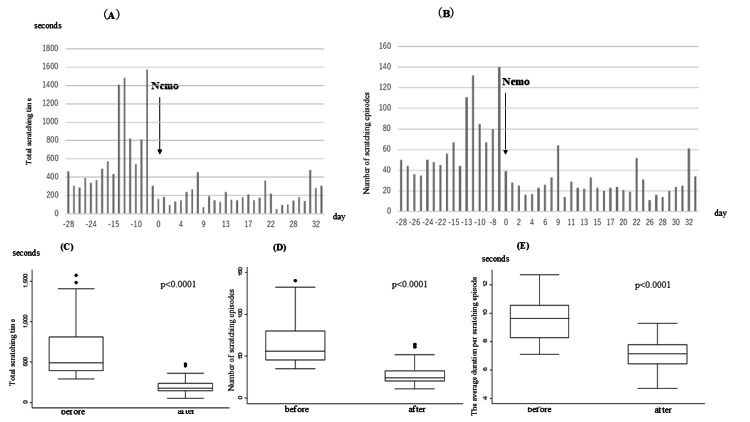
Changes in nocturnal scratching behavior before and after nemolizumab administration (case 2) (A) Daily total scratching time (in seconds) and (B) the number of scratching episodes observed over 28 days before and 35 days after the administration of Nemo (nemolizumab). The day of Nemo initiation is designated as Day 0. Scratching behavior decreased after treatment. (C) Box plots showing the total scratching time, (D) the number of scratching episodes, and (E) the average duration per scratching episode before and after Nemo administration. All metrics significantly improved (p<0.0001)

A 12-year-old male with AD and multiple allergic comorbidities, such as bronchial asthma and cholinergic urticaria, suffered from nocturnal scratching, which was persistent but not self-recognized according to the family, despite intensified topical therapy. He had been treated with topical therapies since early childhood, and during the past year, his treatment mainly consisted of strong-class topical corticosteroids. He had also received oral antihistamine therapy for the management of cholinergic urticaria. One month of monitoring confirmed persistent scratching. He agreed to start dosing of nemolizumab 30 mg, with initial EASI of 13.9 and NRS of 5. At the second dose, EASI was 1.2 and NRS 4. The one-month observation period included several days with transient increases in scratching behavior; however, the severity of these episodes was mild, and overall improvement in scratching behavior was observed (Figure 2).

**Figure 3:**
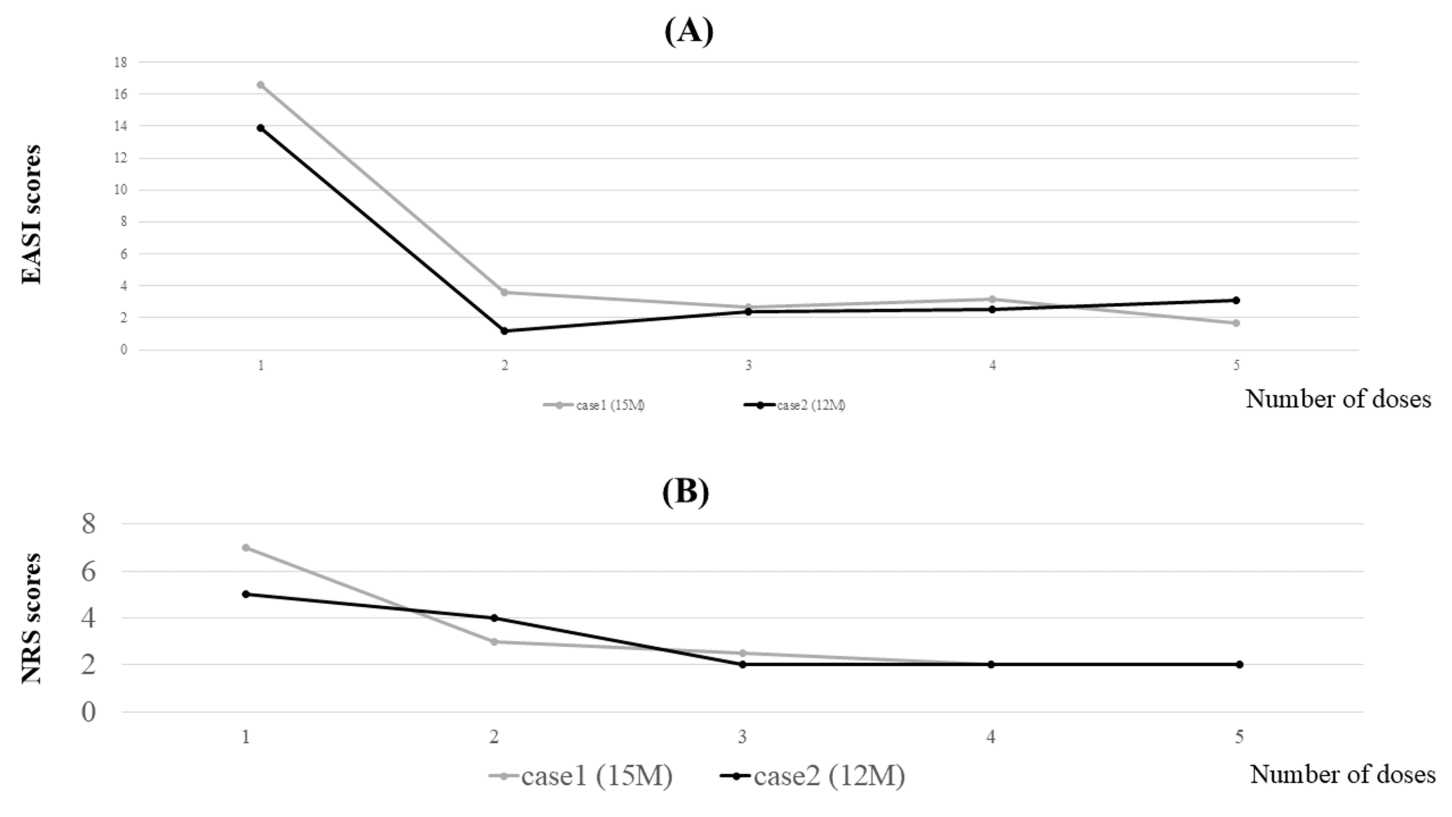
Longitudinal changes in EASI and NRS scores following nemolizumab administration (A) EASI scores and (B) NRS scores over five doses of nemolizumab in two pediatric patients with AD. Although the NRS scores did not reach zero, there was no exacerbation of skin inflammation EASI: Eczema Area and Severity Index; NRS: numerical rating scale

Both patients demonstrated significant improvement in objective scratching behavior and EASI scores after treatment with nemolizumab. Average EASI scores until the fifth dose were 2.8 ± 0.82 (Case 1) and 2.3 ± 0.80 (Case 2), with corresponding NRS scores of 2.38 ± 0.48 and 2.5 ± 1 (Figure 3). Both patients recognized that they had not been sleeping well only after experiencing an improvement in sleep quality following nemolizumab administration, indicating the probable presence of sleep disturbances before treatment initiation. The visualization of scratching behavior bridged the perception gap between patients, families, and physicians, enabling timely and collaborative treatment decisions. In Case 2, nemolizumab treatment did not affect the clinical course of concomitant bronchial asthma or cholinergic urticaria. 

## Discussion

In the present study, we observed a discrepancy between subjective itch assessments and objective scratching behavior in two pediatric patients with AD. As we previously reported [1], the Patient-Oriented Eczema Measure (POEM), a tool for self-assessment of disease severity, did not always correlate with objectively measured nocturnal scratching behavior. Both had experienced chronic itching since infancy, which likely affected their perception of pruritus. Especially in children, subjective measures such as the NRS or VAS may not correctly reflect the true severity of pruritus [1,4,5]. Moreover, physicians tend to estimate itch severity based on visible skin inflammation. However, scratching may persist even in the absence of active lesions, particularly in the presence of psychological stress or comorbidities such as urticaria. These factors contribute to the communication gap between patients and physicians. By visualizing nocturnal scratching behavior with Itch Tracker, we were able to identify potential symptom burden and facilitate more accurate discussions between patients, families, and clinicians. The objective data obtained through the Itch Tracker played a critical role in facilitating SDM [8], which led to the appropriate initiation of nemolizumab in both patients.

This study is the first of its kind to objectively demonstrate a reduction in nocturnal scratching behavior from the first day of nemolizumab administration, with the effect sustained for approximately one month. The data obtained in this study provide valuable insights into the clinical efficacy of nemolizumab and support the appropriateness of a four-week dosing schedule [9,10]. Although the NRS scores did not reach zero and scratching behavior did not completely disappear, the EASI scores showed marked improvement, suggesting that the residual scratching was not severe enough to trigger inflammation. These findings reinforce the notion that pruritus in AD is not solely inflammation-dependent but influenced by diverse contributing factors [2,6,7]. After the administration of nemolizumab, patients often experience a rapid and marked reduction in pruritus in the early phase. Therefore, by the time of the second visit, they may have already adapted to the itch-free state, leading to a self-assessment that was not directly comparable to that at the time of initial dosing.

## Conclusions

Itch Tracker demonstrated its clinical utility by enabling SDM and assessing treatment response in our patients. Objective monitoring of scratching behavior offers a valuable complement to clinical assessments and supports individualized management in pediatric AD.
